# Memories of kuru while at Okapa, Papua New Guinea in 1957

**DOI:** 10.1098/rstb.2008.4026

**Published:** 2008-11-27

**Authors:** Lucy M. Hamilton Reid

**Affiliations:** 25 CliffTerrace, Macleay Island, Queensland 4184, Australia

Before going to Okapa, I had worked in Papua New Guinea (PNG) for 6 years and done nutrition surveys in various parts of the country. The Okapa survey was different because this time I was searching for something unusual in food that could have caused the fatal disease ‘kuru’, so called by the local Fore people owing to the ‘shaking’ that characterizes it. Conducting this study meant that I had to give descriptions and, where possible, collect specimens of everything that was eaten or came into contact with food and send them away for identification. After I had carefully pressed them, I sent my plant specimens to the Lae Herbarium.

At that time, the Okapa people had had very little contact with white people. When being issued with camping gear for an extended survey in this semi-controlled area, I was offered a pistol for protection. I refused it saying, ‘I would be far more frightened of the gun than I would be of the people’. I never had cause to regret my decision.

There were only two permanent European residents at Okapa when I arrived: the Medical Assistant in charge of the Native Hospital and the Patrol Officer Jack Baker. All buildings were made of local materials except for the Patrol Officer's timber house where I was billeted. Dr Vincent Zigas, the government Medical Officer for the district, who discovered kuru as an epidemic disease, had his home in Kainantu, but visited frequently. An American, Dr Carleton Gajdusek, who was researching this strange disease that could not then be identified, also stayed in Jack Baker's house (figure 1) when he was not on patrol. A new hospital constructed with local materials had recently been erected at Okapa for the investigation and treatment of kuru patients.

It was decided that I should do my survey in Moke village, where many kuru sufferers had been found. Moke was situated on a ridge across a deep ravine from Okapa station. The two settlements were within sight of each other but 2 hours' walking distance apart. A new house was built for me at Moke. It was a little different from the usual village houses in that it had a window and a roof that permitted me to stand upright when inside. Cooking was done on an outside fire.

Most people spoke only their own language (Fore) but a few younger ones knew Tok Pisin (Melanesian Pidgin). I employed two girls and a boy as interpreters plus a young man as cook. On my first afternoon in the village, I went for a walk in a garden and startled an old woman. She was so surprised because she had never seen a white person before. After being reassured that I would not harm her, she came close and felt me, then asked through my interpreter whether I had been born in the usual manner or had just arrived on Earth like that.

To carry out investigations, I went of a morning to the gardens with the women and children. Specimens of plants used as food, condiments, medicines and wrappings for food and leaves used for lining and covering mumus (earth ovens in which cooking was done over hot rocks; figures 2 and 3) were brought to me.

I was also brought leaves from the many types of forest trees used for making salt. To make salt, a huge pile was made of branches and leaves from special trees. The pile would be left until dry, then set alight and allowed to burn until only ash remained. The salt was leached out of the ash and the solution evaporated to produce a cake of salt, which tasted to me like potassium salt (which indeed it was).

Later in the day, I would go to the houses and watch women preparing the evening meal, and visit their houses again when they were eating their meal. Because I was so interested in their food, the people were also interested in what I ate. For the first few days I was in the village, everybody seemed to come and crowd into my house to watch me eat my evening meal. After a little while the adults would slip away, but some of the children would stay well into the night. There were stacks of newspapers and magazines for pressing plant specimens. I showed the children pictures in them and tried to explain the outside world.

One night while the children looked at pictures, I read a book. When I laughed out loud at some amusing passages it puzzled the children. They looked at my book. There were no pictures, and they did not comprehend that printing meant anything, so they thought I was laughing at nothing like kuru sufferers! They hurried away and got their parents, who came and watched me for a short while and then left, leaving the children behind. No doubt they considered me harmless even if a bit mad.

I went on a week-long patrol with Carleton Gajdusek, Vin Zigas, Jack Baker and visitor Lois Larkin. Lois was a laboratory assistant with Sir Macfarlane Burnet in Melbourne and had come to work with Carleton; she later married Jack. The purpose of the patrol was to find out how far kuru had spread by searching for it in outlying areas. Most of the people in these parts had never seen a white woman and only rarely a white man. Carriers took our food and camping gear, but even without anything to carry I knew my short stature would make it impossible for me to keep up with the party, so I employed three young boys to assist me up the steep, often slippery tracks. With a boy pulling on each arm and the third pushing my backside, I managed not to be left behind. Two of these boys (now men of retiring age) attended the End of Kuru Conference in London and I was privileged to be photographed there with them. Occasionally, on the roads near Okapa station, I was able to travel more comfortably by other means (figure 4).

While we were on patrol, we slept at night in village houses. About dusk one day, we came to a village where some old men were returning from the gardens. The men of our party began negotiating with them about staying there that night; then the young women with their bundles of food and babies arrived and stood a little way apart. I went to speak with them as best I could. They knew no Pidgin and I none of their language, but they understood when I admired their babies. Then out of the growing darkness came a young man covered in pig grease and sweat, carrying a bow and arrows. Wearing only a g-string and the traditional bunch of leaves at the back, he had a pig tusk through his nose and hair comprising lots of greasy plaits. He stood close by watching us for a few minutes. Suddenly, he picked me up, threw me over his shoulder and began purposefully striding towards the forest. I thought not to scream, else he might run for the forest before being stopped. There it would be difficult to find me in the dark. As soon as he reached the forest edge, I screamed, knowing that the women would make a hullabaloo—which they did. This attracted the attention of the men, who called to my abductor to stop. He did so, abruptly releasing me. I slid to the ground only several strides from the forest. What could have been a nasty incident, especially for me, was passed off as a joke, and we were treated with respect thereafter and spent a peaceful night in the village.

My research showed that the Fore people ate similar foods to those eaten by other groups in the PNG highlands. Anthropologists who came before and after me described their cannibalistic practices. In 1957, they were living as they had done for thousands of years. They had no cooking pots or implements from outside their area, except for a very few steel knives and axes, which were prized possessions. A great abundance of leaves from many kinds of tree and smaller plants were used to wrap food for mumu cooking or to use as plates. Pieces of bark or wooden plates were sometimes used when preparing food or to place food on to cool after cooking.

Their eating pattern was as follows. In the morning cold sweet potato left over from the evening meal was taken and eaten while going about their morning duties. During the day, snacks of fruit found in the garden or forest might be eaten, but this was not considered real food. Occasionally, wild creatures such as rats, possums or birds might be caught, cooked and eaten at once, or taken home for the evening meal. The children often caught small animals or insects that they cooked and ate on their own.

The evening meal consisted mainly of sweet potato cooked after dark in the coals of the house fire. Many leafy green vegetables obtained from the gardens and nearby forest were consumed. They were crammed into hollow bamboo and cooked in the embers, being turned frequently to cook evenly and prevent the bamboo cylinder burning. The meal was a social event, visitors often dropping in. The members of the household would eat and chat well into the night. They did not rise in the morning until the sun was well up, the day quite warm.

The Fore differed from some other highland people in that they ate pigs more frequently. In many other areas of the highlands, pigs were eaten only at feasts or ceremonies. In Moke village, a pig was cooked in a mumu almost every week. Other families would be invited and they contributed bundles of different vegetables to be cooked with the pig. These practices gave the Fore more regular protein, hence a better diet.

Overall, in my work, I indispensably disproved some of the negative ‘causes’ of kuru, thus enabling Dr Carleton Gajdusek to score his deserved Nobel Prize!

## Figures and Tables

**Figure 1:**
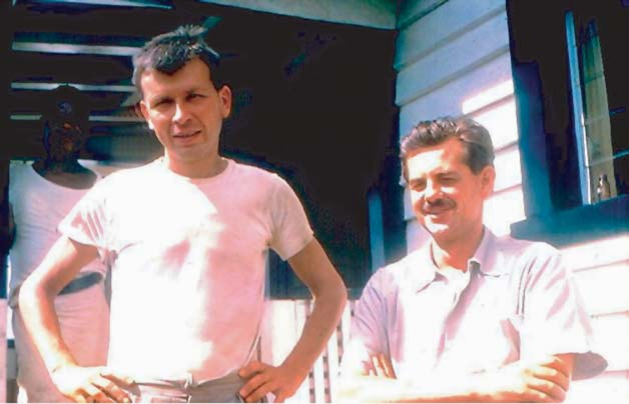
Carleton Gajdusek and Jack Baker outside Jack's house in Okapa in 1957.

**Figure 2:**
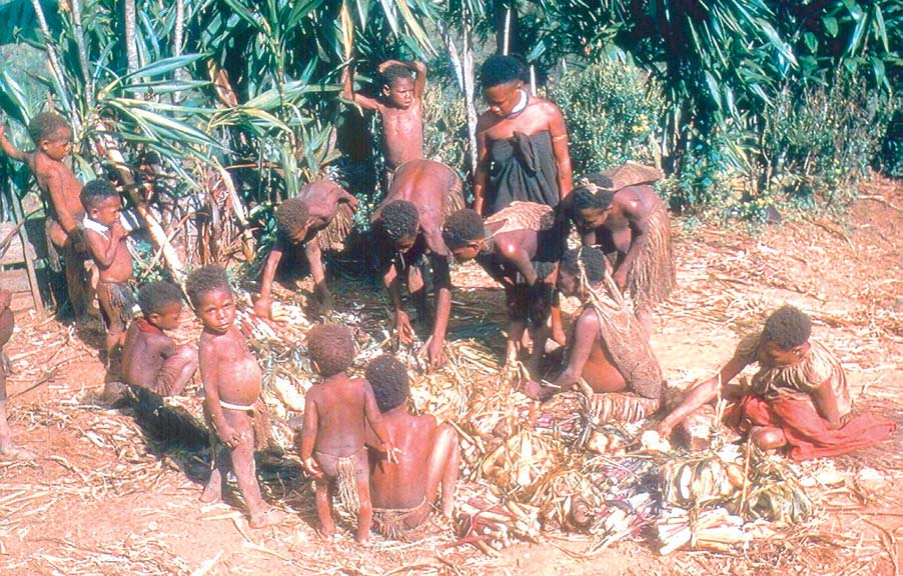
Moke village women preparing food for cooking in a mumu; the bundles of food in the front are of edible highlands pitpit; other bundles contain sweet potato, the dietary staple of the region.

**Figure 3 fig3:**
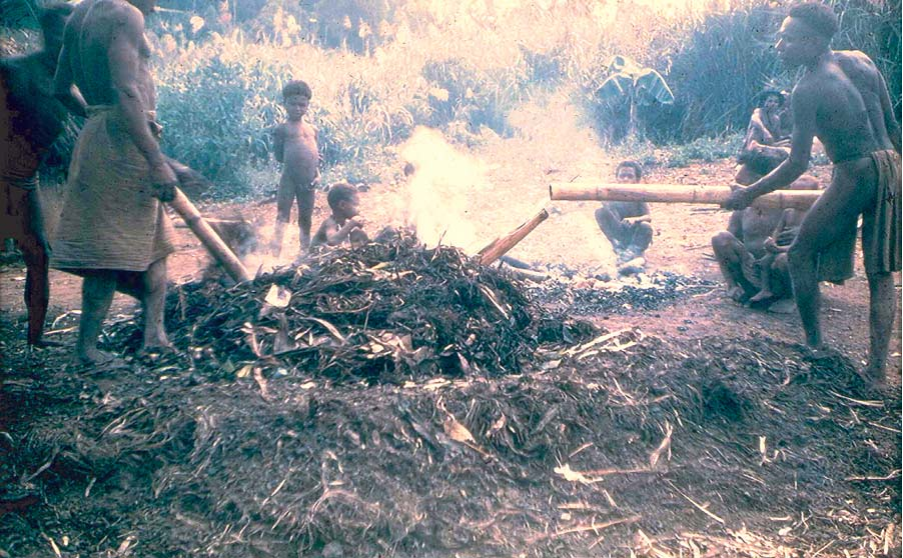
Completing the earth oven of the mumu: after the hot stones have been placed in the pit they are covered and the pit is lined with leaves; the bundles of food are placed inside the pit on top of the stones and covered with leaves; once the mumu has been sealed (often with earth), water from bamboo cylinders is poured in so as to reach the hot rocks below and generate steam.

**Figure 4:**
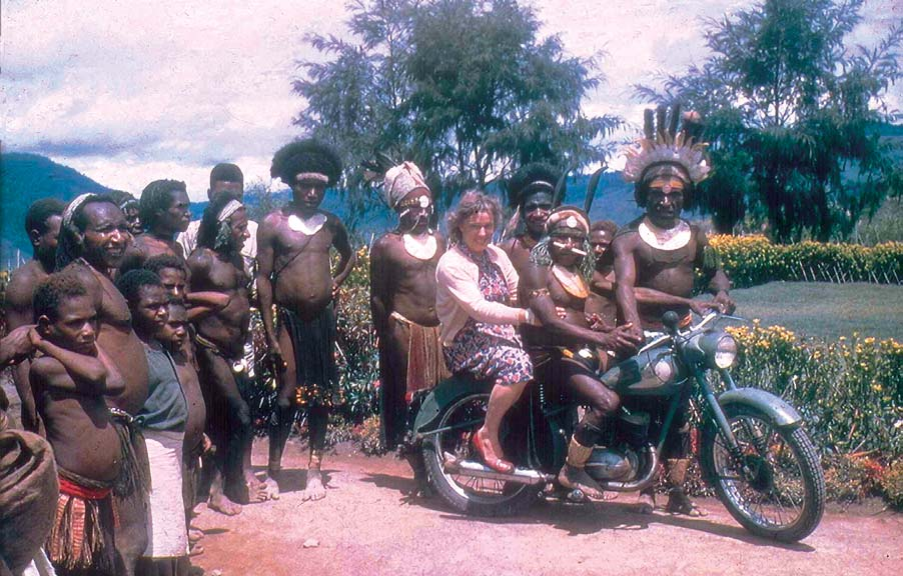
I am ready to take off from Okapa station, but not to go far, in 1957.

